# Effect of pegloticase on renal function in patients with chronic kidney disease: a post hoc subgroup analysis of 2 randomized, placebo-controlled, phase 3 clinical trials

**DOI:** 10.1186/1756-0500-7-54

**Published:** 2014-01-21

**Authors:** Robert A Yood, Faith D Ottery, William Irish, Marsha Wolfson

**Affiliations:** 1 Reliant Medical Group, 630 Plantation Street, Worcester, MA 01605, USA; 2 Savient Pharmaceuticals Inc., 400 Crossing Boulevard, 3rd Floor, Bridgewater, NJ 08807, USA; 3 CTI Clinical Trial and Consulting Services, 8380 Six Forks Road, Suite 203, Raleigh, NC 27615, USA; 4 Reliant Medical Group, 425 North Lake Ave., Worcester, MA 01605, USA

**Keywords:** Refractory chronic gout, Uric acid, Kidney, Renal function

## Abstract

**Background:**

Pegloticase is approved in the US for treatment of refractory chronic gout. Since chronic kidney disease (CKD) is common in these patients, we conducted a post-hoc analysis of 2 replicate phase 3 trials and the subsequent open-label extension study to determine the effects of pegloticase on renal function in patients with CKD stages 3 and 4, as well as the effects of renal dysfunction on pegloticase efficacy and safety.

**Findings:**

Patients with renal insufficiency were randomized to pegloticase 8 mg every 2 weeks (n = 42), pegloticase 8 mg every 4 weeks (n = 41), or placebo (n = 20) for 6 months as defined by the study protocols. Renal function was assessed by estimated glomerular filtration rate (eGFR). All patients completing the randomized trials could participate in an open-label extension study for a further 2.5 years. Uric acid response, the primary end point in the trials, was plasma uric acid <6.0 mg/dl for 80% of months 3 and 6.

Mean eGFR in both pegloticase dosing cohorts remained constant over the randomized treatment phase and long-term open-label extension study. The number of patients achieving uric acid response was similar across CKD stages (32% stage 1, 23% stage 2, 35% stage 3, and 39% stage 4, respectively, P = 0.3). There was no difference in the pegloticase safety profile based on CKD stage.

**Conclusions:**

Pegloticase treatment does not impact eGFR in CKD patients and response to pegloticase is independent of CKD stage.

**Trial registration:**

Clinical trial identifier: NCT00325195

## Findings

### Background

Pegloticase is a novel recombinant, mammalian uricase covalently linked to monomethoxypolyethylene glycol, and is approved in the United States for treatment of refractory chronic gout (RCG) [1-4]. Pegylation enables the conjugate to have a prolonged elimination half-life of up to 14 days following intravenous infusion [4]. Pegloticase rapidly degrades plasma urate to allantoin, a more soluble compound that is easily excreted by the kidneys [2-4]. Due to the low plasma urate level, extravascular urate moves into the plasma compartment down its concentration gradient where it is available for degradation by pegloticase. Among patients who respond to therapy, this process is believed to engender urate crystal dissolution within soft tissue and peri-articular structures, with eventual normalization of body urate stores, and resolution of RCG signs and symptoms [3-6].

The prevalence of gout is highest in individuals >60 years of age [7], who also often have chronic kidney disease (CKD) [8,9]. Indeed, results of a recent retrospective database analysis indicated that a large proportion (approximately 40%) of patients with gout had CKD [10]. While gout and CKD are likely comorbidities with a shared etiology, causative mechanistic factors have yet to be elucidated. It is known, however, that CKD is an independent risk factor for chronic gout [11,12] and, vice versa, that hyperuricemia and gout are risk factors for CKD [13-18]. From a clinical practice perspective, CKD limits the use of some urate-lowering therapies and complicates symptomatic treatment with nonsteroidal anti-inflammatory drugs and colchicine [19], although colchicine remains a viable treatment option for acute gout providing dosage reductions are made for moderate-to-severe renal impairment [20,21].

We conducted a post-hoc subgroup analysis to determine the effects, if any, of pegloticase on renal function in patients with CKD stages 3 and 4 (estimated glomerular filtration rate [eGFR], 30–59 ml/min/1.73 m^2^ and 15–29 ml/min/1.73 m^2^, respectively [9]). Conversely, we also evaluated the effects renal dysfunction may have on pegloticase efficacy and safety.

## Methods

### Summary of study design and entry criteria

Data were retrospectively analyzed from 2 randomized, placebo-controlled, replicate phase 3 clinical trials (n = 212) of pegloticase use over 6 months in patients with RCG to determine the effects of pegloticase on renal function. A total of 49% of patients from the randomized controlled trials (RCTs) met the criteria for stages 3 and 4 CKD [9]. Since the designs of the phase 3 trials were identical, data were pooled to enable an integrated comparison of the treatment regimens. The effect of long-term pegloticase treatment on renal function was evaluated using data from patients with CKD from the RCTs who entered the open-label extension (OLE) study.

The full methodology of the 2 randomized, double-blind, multicenter, placebo-controlled trials (C0405 and C0406; NCT00325195) has been published previously [6]. Briefly, patients were ≥18 years of age and had RCG based on a baseline serum uric acid of ≥8.0 mg/dl and ≥1 of the following: ≥3 self-reported gout flares during the previous 18 months; ≥1 tophi; and gouty arthropathy (defined clinically or radiographically as joint damage caused by gout). Patients also had contraindication to treatment with allopurinol or history of failure to normalize uric acid despite ≥3 months of treatment with the maximum medically appropriate allopurinol dose [6]. Patients receiving dialysis were excluded. Both studies were conducted in accordance with the ethical principles of the Declaration of Helsinki and received institutional review board approval at each site (local) or by a central institutional review board (IntegReview, Austin, Texas). All patients gave written, informed consent [6].

### Treatment regimens

Patients in the RCTs were assigned in a 2:2:1 ratio to receive intravenous infusions of pegloticase 8 mg every 2 weeks, pegloticase 8 mg every 4 weeks (alternating with placebo to maintain blinding), or placebo for 24 weeks. After completing the study, subjects were given the option of continuing active treatment for up to 2.5 years through a follow-on OLE study. Dose adjustment was permitted twice during the OLE study.

### Renal function

The primary end point in this post-hoc analysis was the impact of up to 6 months of pegloticase on renal function in patients with CKD stages 3 and 4, as defined by the National Kidney Foundation Kidney Disease Outcomes Quality Initiative [9]. Renal function was assessed by eGFR using the 4-variable Modification of Diet in Renal Disease (MDRD) formula: eGFR (ml/min/1.73 m^2^) = 186 × (SCr)^-1.154^ × (age)^-0.203^ × (0.742 if female) × (1.212 if black/African American), where SCr is serum creatinine in mg/dl and age in years [22]. eGFR was calculated at screening (week 0) and at protocol-defined time points (weeks 7, 13, 19, and 25 post-randomization). For patients who participated in the OLE study, eGFR was calculated at weeks 13, 25, 37, 53, 65, 77, 89, and 101 following start of treatment in the OLE treatment phase. CKD stage was based solely on the eGFR calculation.

Absolute and relative changes from baseline (screening) in eGFR were calculated as follows:

(1) Absolute change = minimum eGFR post-screening – screening eGFR.

(2) Percent change = 100 × (minimum eGFR post-screening – screening eGFR)/screening eGFR.

(3) Percent change in eGFR was further categorized as follows: Category 1, no change (Δ) or improvement; Category 2, 0 < Δ ≤ 10%; Category 3, 10 < Δ ≤ 20%; Category 4, 20 < Δ ≤ 30%; Category 5, 30 < Δ ≤ 40%; Category 6, 40 < Δ ≤ 50%; and Category 7, Δ > 50%.

### Responders

Uric acid response was the primary end point in the pegloticase phase 3 trials. A responder was defined as a patient with plasma uric acid level <6.0 mg/dl for 80% of the time during months 3 and 6 combined.

### Analysis population

Clinical outcomes were assessed in the modified intention-to-treat population (mITT), defined as all patients with RCG who received ≥1 infusion of study medication.

### Statistical analysis

Continuous data are presented as the mean ± standard deviation (SD) or median and range, and categorical data as counts and percentages. A linear mixed effects (random intercept) model was used to analyze longitudinal measures of eGFR assuming an unstructured covariance matrix for the random intercept effect [23].

Treatment (i.e., pegloticase every 2 weeks and every 4 weeks versus placebo), visit (week), treatment × visit, age (years), sex (female versus male) and race/ethnicity (black/African American, Hispanic/Latino and other versus white) were included as fixed effects, while subject was included as a random effect. Two-way interactions of treatment with age, sex, and race/ethnicity were tested to evaluate whether the overall impact of treatment was differentially affected by age, sex, or race/ethnicity. Absolute and percentage change in eGFR was compared between groups using general linear model adjusted for age, sex, race/ethnicity, and responder status.

## Results

### Study populations

Baseline eGFR and CKD data for the full population of the RCTs is shown in Table 1. Among these patients, 103 (49%) had CKD stage 3 (n = 80) or 4 (n = 23); no patients had CKD stage 5 by protocol exclusion. Forty-two of 103 patients (41%) received pegloticase 8 mg every 2 weeks, 41 patients (40%) received pegloticase 8 mg every 4 weeks, and 20 patients (19%) received placebo.

**Table 1:** CKD stage definition and summary overall and by randomized treatment group per clinical trial*

		**Study CO405 (%)**				**Study CO406 (%)**			
**CKD stage**	**eGFR definition**^**†**^	**PGL q2wks**	**PGL q4wks**	**Placebo**	**Overall**	**PGL q2wks**	**PGL q4wks**	**Placebo**	**Overall**
		**(n = 43)**	**(n = 41)**	**(n = 20)**	**(n = 104)**	**(n = 42)**	**(n = 43)**	**(n = 23)**	**(n = 108)**
1	≥90	8 (18.6)	6 (14.6)	3 (15.0)	17 (16.3)	9 (21.4)	5 (11.6)	3 (13.0)	17 (15.7)
2	60-89	14 (32.6)	12 (29.3)	4 (20.0)	30 (28.8)	12 (28.6)	19 (44.2)	13 (56.5)	44 (40.7)
3	30-59	15 (34.9)	19 (46.3)	11 (55.0)	45 (43.3)	17 (40.5)	13 (30.2)	5 (21.7)	35 (32.4)
4	15-29	6 (14.0)	4 (9.8)	2 (10.0)	12 (11.5)	4 (9.5)	5 (11.6)	2 (8.7)	11 (10.2)
5	<15 (or dialysis)	0	0	0	0	0	0	0	0

CKD, chronic kidney disease; eGFR, estimated glomerular filtration rate; PGL, pegloticase; q2wks, every 2 weeks; q4wks, every 4 weeks.

*Based on the intent to treat analysis data set; that is, all randomized patients who received at least one dose of treatment.

^†^As described by the National Kidney Foundation Kidney Disease Outcomes Quality Initiative.

Baseline characteristics were comparable across the CKD stage 3 and 4 cohort for each of the 3 study arms. For the full CKD stage 3/4 cohort, mean age was 61.7 years, 71% were men, 67% were white, and 18% were black or African American. Mean eGFR at screening was 40.4 ml/min/1.73 m^2^ and 40.1 ml/min/1.73 m^2^ in the pegloticase biweekly and monthly arms, respectively, versus 43.3 ml/min/1.73 m^2^ in the placebo arm (P = 0.6). For the complete RCT population (n = 212) mean age was 55.4 years, 81.6% were men, 67.5% were white, and 13% were black.

Of the 103 patients with CKD stage 3 or 4 in the RCTs, 76 (74%) participated in the OLE study. Two patients received no pegloticase treatment and were excluded from further analysis, 43 (58%) received pegloticase 8 mg every 2 weeks, and 31 (42%) received pegloticase 8 mg every 4 weeks. No statistically significant difference in patient demographics was observed between treatment groups entering the OLE study. Mean age was 62 years, 65% of patients were men, and 70% of patients were white.

### Phase 3 clinical trials

**Figure 1:**
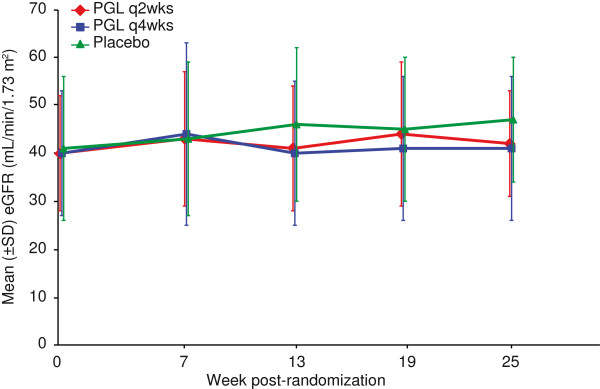
**Observed mean eGFR (± SD) over time by treatment group in the RCTs.** Mean eGFR was determined every 6 weeks for patients in each of the randomized treatment groups (pegloticase infused every 2 weeks, pegloticase infused every 4 weeks alternating with placebo, and placebo) during the 6-month trials. PGL = pegloticase, SD = standard deviation, eGFR = estimated glomerular filtration rate.

**Figure 2:**
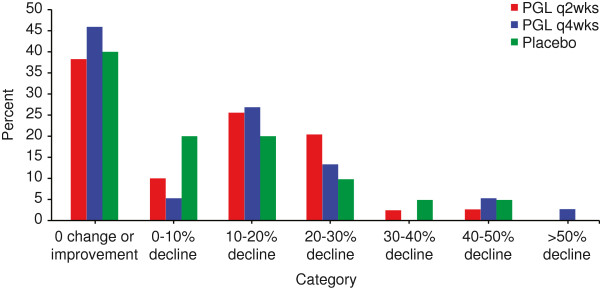
**Percentage of patients with a change in eGFR by categories of change.** Change was calculated as the percentage change from baseline in the minimum eGFR observed at post-randomization visit. Categories of change were derived as a proxy for severity of renal function decline. Percentages are presented by randomized treatment group. PGL = pegloticase.

Mean eGFR remained constant in the 3 study arms over the 25-week randomized treatment phase (Figure 1). Change in eGFR was not differentially affected by treatment (treatment × time interaction: P = 0.277), independent of age, sex, and race/ethnicity. In addition, no discontinuation pattern was observed that may have biased the treatment effect toward the null hypothesis of no treatment difference. Mean absolute and percentage changes from baseline in eGFR for individuals were consistent with overall findings from the linear mixed effects model (Table 2 and Figure 2). More than one-third of patients in all groups had either no change or an improvement in renal function during the 25-week randomized treatment phase, and approximately one-half of patients in all groups had no more than a 10% decline in renal function (Figure 2).

**Table 2:** Absolute and percentage change in eGFR in patients with CKD stages 3 and 4

**Parameter**	**PGL q2wks (n = 42)**	**PGL q4wks (n = 41)**	**Placebo (n = 20)**	P value*
**Absolute change in eGFR**				
Mean (SD)	−2.8 (8.2)	−1.1 (11.2)	−2.7 (8.1)	0.607
Median (min, max)	−4.12 (−19.6, 19.7)	−2.63 (−15.2, 54.6)	−2.54 (−18.6, 16.1)	
**Percent change in eGFR**				
Mean (SD)	−4.2 (24.5)	−4.4 (27.3)	−5.8 (21.2)	0.853
Median (min, max)	−10.4 (−45.5, 94.2)	−9.6 (−57.7, 106.6)	−6.1 (−46.0, 52.3)	

eGFR, estimated glomerular filtration rate; CKD, chronic kidney disease; PGL, pegloticase; q2wks, every 2 weeks; q4wks, every 4 weeks; SD, standard deviation.

*A general linear model F-test, adjusted for age, sex, and race/ethnicity, was used to assess the overall impact of treatment.

No difference was detected in responder rates to pegloticase therapy by CKD stage. The percentages of responders in subgroups with CKD stage 1, stage 2, stage 3, and stage 4 were 32%, 23%, 35%, and 39%, respectively, (P = 0.3). Responders had statistically significant lower mean eGFR compared with nonresponders at baseline (59.1 ± 25.3 ml/min/1.73 m^2^ versus 63.3 ± 24.6 ml/min/1.73 m^2^) and at 6 months (61.5 ± 24.6 ml/min/1.73 m^2^ versus 63.8 ± 25.1 ml/min/1.73 m^2^; P = 0.026).

### OLE study

Mean (± SD) eGFR at week 1 of the OLE study (after a gap in therapy between trials) was 41.9 ± 14.0 ml/min/1.73 m^2^ for patients who received pegloticase 8 mg every 2 weeks and 45.3 ± 12.4 ml/min/1.73 m^2^ for patients who received 8 mg pegloticase every 4 weeks (P = 0.299). Total mean (± SD) treatment duration (from RCT randomization) was also similar between the pegloticase 8 mg every 2 weeks and pegloticase 8 mg every 4 weeks cohorts (88 ± 33 weeks [range, 8–123 weeks] versus 84 ± 33 weeks [range, 12–123 weeks], respectively, P = 0.7).

Mean (± SD) eGFR at week 101 in the OLE study was 46.0 ± 13.3 ml/min/1.73 m^2^ in patients receiving pegloticase biweekly compared with 40.6 ± 14.9 ml/min/1.73 m^2^ in patients receiving pegloticase monthly (P = 0.3). No change in eGFR was detected with respect to time (treatment × time interaction: P = 0.9), independent of age, sex, and race/ethnicity.

### Safety and tolerability

When assessed for the full safety population of patients in the RCTs, gout flares and infusion reactions were the 2 most common adverse events. Multiple subgroup analyses from the RCTs have confirmed this finding. Further, there were no differences in the pegloticase safety profile based on CKD stage and no new safety signals were detected in this cohort.

## Discussion

This post-hoc subgroup analysis has revealed several new findings of relevance to clinical management of RCG. First, patients with stage 3 or 4 CKD had no clinically meaningful changes in renal function with up to 6 months of pegloticase therapy. Similarly, no changes in renal function were observed in patients who participated in the long-term OLE study for a total mean period of 1.5 years of pegloticase therapy. Second, pegloticase efficacy and safety were not affected by renal function status. Finally, response to pegloticase was independent of CKD stage. While there was a statistically significant trend indicating that responders had lower eGFRs than nonresponders, change in eGFR over time was not different as a function of responder status.

One strength of the present analysis is the utilization of prospectively collected data derived from randomized clinical trials that featured a placebo arm. However, the analysis is limited by established drawbacks of retrospective data analyses. In addition, the phase 3 clinical trials were not powered to detect changes in renal function across randomized treatment groups.

Normalization of hyperuricemia for extended periods in patients with renal impairment may have clinical implications. Urate-lowering with allopurinol and febuxostat has been associated with preservation of renal function in patients with gout; results reported here for pegloticase are consistent with these previous findings [24-26]. While no change or decrement in mean eGFR was associated with pegloticase therapy during the randomized and OLE studies, the slow rate of progression of CKD means that a larger sample size must be followed for a longer period of time to confirm this observation. Rarely, hypouricemia resulting from mutations in URAT1 and GLUT9 has been associated with exercise-induced acute kidney injury. Mechanistically this relationship is poorly understood [27-29] and it should be noted that pegloticase has no effect on urate/anion transporters. One patient in the pegloticase trials developed acute kidney injury attributed to sepsis and chronic renal insufficiency with no evidence for a relationship with hypouricemia.

## Conclusion

In conclusion, these data provide support for the use of pegloticase in the management of patients with RCG and comorbid CKD stages 3 and 4 at the approved dosage of 8 mg every 2 weeks.

## Abbreviations

RCG: Refractory chronic gout; CKD: Chronic kidney disease; eGFR: estimated glomerular filtration rate; RCT: Randomized controlled trial; OLE: Open-label extension; MDRD: Modification of Diet in Renal Disease; SCr: Serum creatinine; mITT: modified intention-to-treat; SD: Standard deviation.

## Competing interests

William Irish has received research support from Novartis and Genentech and is a consultant for Tolera. Faith Ottery is a previous employee of Savient Pharmaceuticals, Inc. Marsha Wolfson is a previous employee and currently receives consulting fees from Savient Pharmaceuticals, Inc. Robert Yood has received grant support from Takeda Pharmaceuticals.

## Authors’ contributions

FO and RY were involved in the design, implementation, and data analysis for the phase 3 trials. MW provided clinical guidance on the renal function analysis and data interpretation. BI performed the renal function data analysis. All authors helped to draft the manuscript, provided guidance on revisions, and approved the final manuscript.

## Authors’ information

Marsha Wolfson is formerly of Savient Pharmaceuticals Inc.
